# VCformer: Variable-Centric Multi-Scale Transformer for Multivariate Time Series Forecasting

**DOI:** 10.3390/s25165202

**Published:** 2025-08-21

**Authors:** Junyu Zhu, Enguang Zuo, Xinyu Bi, Chen Chen, Cheng Chen, Ziwei Yan, Xiaoyi Lv

**Affiliations:** 1 School of Computer Science and Technology, Xinjiang University, Urumqi 830046, China; 107552304224@stu.xju.edu.cn (J.Z.); 107552401284@stu.xju.edu.cn (X.B.); 2 School of Intelligence Science and Technology, Xinjiang University, Urumqi 830017, China; zeg@xju.edu.cn; 3 Department of Electronic Information, Tsinghua University, Beijing 100084, China; 4 School of Software, Xinjiang University, Urumqi 830091, China; chen_chen@stu.xju.edu.cn (C.C.); chenchengoptics@gmail.com (C.C.); ziwei13579@163.com (Z.Y.)

**Keywords:** multivariate time series, long-term sequence forecasting, sequence transposition, representation learning

## Abstract

Multivariate time series forecasting is crucial for numerous practical applications ranging from financial markets to climate monitoring. Traditional multivariate time series forecasting methods primarily adopt a time-centric modeling paradigm, applying attention mechanisms to the temporal dimension, which presents significant limitations when handling complex dependencies between variables. To better capture inter-variable interaction patterns, this paper proposes the Variable-Centric Transformer (VCformer), which shifts the attention paradigm from time-centric to variable-centric through sequence transposition. Building upon this foundation, we further design a dual-scale architecture that simultaneously models feature representations at both the original variable level and variable group level. Combined with an adaptive variable grouping mechanism, we construct a parameter-sharing dual-path encoder and finally select the optimal feature fusion strategy through comparative experiments. Experimental results on seven benchmark datasets demonstrate that VCformer achieves comprehensive improvements in prediction accuracy compared to traditional time-centric methods, while exhibiting stronger modeling capabilities on high-dimensional data. Ablation studies and interpretability analysis further validate the effectiveness of each component.

## 1. Introduction

**Background:** Multivariate time series forecasting is an important research direction in time series analysis [1], with widespread application demands in fields such as financial market analysis [2], weather forecast [3,4], energy system management [5], and intelligent traffic control [6]. Unlike univariate time series forecasting, multivariate time series data contains multiple interrelated variables that not only possess individual temporal evolution characteristics, but more importantly, exhibit complex interdependencies among them. Traditional multivariate time series forecasting methods mostly adopt time-centric modeling paradigms. Whether they use classical statistical methods [7] or deep learning-based approaches such as LSTM [8] and GRU [9], they primarily focus on sequential dependencies in the temporal dimension. Recent Transformer-based [10] time series forecasting methods, while excelling at capturing long-range temporal dependencies, continue this paradigm by mainly employing time-centric attention mechanisms that treat each time step as the basic computational unit.

**Figure 1:**
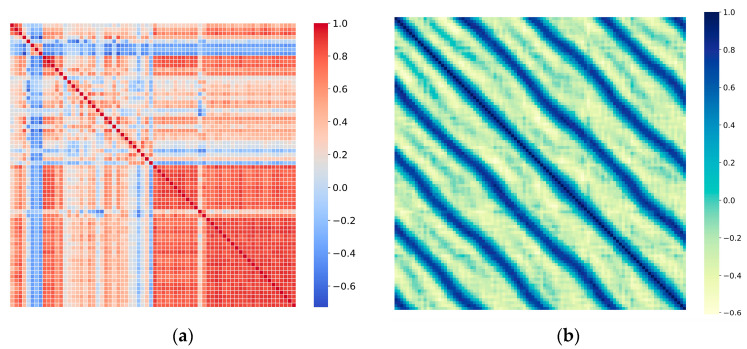
(**a**) An inter-variable correlation heatmap showing the pairwise relationships between different variables in the multivariate time series. The color intensity represents the correlation strength, with red indicating positive correlations and blue indicating negative correlations. The clear block structures reveal distinct variable groups with strong internal correlations. (**b**) An intra-variable temporal attention heatmap displaying the temporal attention patterns within variables. The diagonal stripe patterns indicate the model’s ability to capture both local temporal dependencies and long-range relationships across different time steps.

**Challenge:** Based on the above background, we analyzed the correlations in time series along both temporal and variable dimensions, observing that the following: (1) Variables exhibit block structures within the same timestamp, as shown in Figure 1a, reflecting dynamic dependencies between variables. (2) The same variable demonstrates clear trends and periodic fluctuations over time periods, as shown in Figure 1b. We further analyzed the model’s focus on time series features under different feature embedding approaches. In classical embedding methods, sequences are processed along the channel dimension with each time point’s multivariate components treated as a token, which has an obvious deficiency: it obscures inter-variable dependencies. In contrast, transposed embedding approaches perform dimension transposition on sequences and treat each variable independently as a token, which to some extent alleviates the deficiency of blurred variable relationships, enabling the model to learn inter-variable dependencies while preserving temporal dependencies. Therefore, the time-centric paradigm has inherent limitations when handling complex inter-variable relationships. While this approach is effective for sequential modeling, it cannot fully utilize the complex inter-variable relationships inherent in multivariate systems. In multivariate systems, interactions between variables are often more complex and important than temporal dependencies. Consider a weather forecasting scenario involving variables such as temperature, humidity, atmospheric pressure, and wind speed. The relationships between these variables are typically more predictive than their individual temporal patterns [11]. Furthermore, in stock markets, correlations between different stocks may be more decisive for future trends than individual stock historical patterns [12]; in meteorological systems, spatial correlations between meteorological elements at different geographical locations are often key to prediction [13]. How to effectively model these inter-variable interaction patterns is the core challenge facing multivariate time series forecasting. Traditional time-centric methods struggle to directly model these complex inter-variable interaction patterns, treating inter-variable relationships merely as additional constraints or implicitly modeling them through parameter sharing [14], which stems from the modeling complexity brought by the multivariate characteristics of time series data.

**Solution:** To address the above issues, this paper proposes a novel modeling paradigm based on Variable-Centric Multi-scale Transformer (VCformer). Our core insight is that inter-variable interaction relationships are usually more informative than individual variable temporal patterns for multivariate time series forecasting. Our core innovation is converting the traditional time-centric paradigm to variable-centric modeling and designing a dual-scale architecture on this basis to comprehensively capture multi-level variable interaction patterns. Specifically, given multivariate time series data $X\in {\mathbb{R}}^{T\times N}$, where T is the time length and N is the number of variables, traditional time-centric methods treat it as a sequence of T time steps, each containing observations of N variables. Our method reorganizes it into a collection of N variables through sequence transposition, where each variable contains complete historical information of T time steps, making each variable’s complete time series a modeling unit (token), then applying attention mechanisms in the variable dimension to directly learn interaction patterns between any two variables. To fully exploit the complexity of inter-variable relationships, we observe that inter-variable interactions often manifest at multiple levels: both direct inter-variable relationships and higher-level collaborative patterns between variable groups. Therefore, we design a dual-scale architecture that simultaneously processes representations at two different granularities through dual encoders: the original scale directly applies variable-centric attention mechanisms to N original variables, learning fine-grained direct inter-variable interaction relationships; the grouped scale organizes N variables into several variable groups through a learnable variable grouping strategy, then applies attention mechanisms at the group level to capture coarse-grained inter-group collaborative patterns. This dual-scale design enables the model to simultaneously capture multi-level variable interaction patterns from microscopic to macroscopic scales, discovering higher-order variable combination relationships while preserving the complete time series information of original variables. Additionally, in high-dimensional multivariate systems, the number of variables is typically much smaller than the length of the time series. This design effectively reduces attention computation complexity while ensuring that the learned inter-variable attention weights possess clear physical meaning, significantly enhancing model interpretability. The main contributions are as follows:

- Proposes a variable-centric modeling paradigm that achieves fundamental transformation from time-centric to variable-centric through sequence transposition techniques, providing a novel modeling approach for multivariate time series forecasting.
- Designs a dual-branch processing architecture for original variables and grouped variables, simultaneously capturing fine-grained variable-level and coarse-grained group-level patterns through a dual-encoder architecture, demonstrating stronger expressive capability compared to single-scale methods.
- Adaptive Grouping Mechanism: Proposes a learnable variable grouping strategy that can automatically discover variable association patterns in data without requiring prior knowledge.
- Demonstrates superior performance on eight benchmark datasets, achieving optimal balance between computational efficiency and modeling capability through comparative analysis of multiple dual-scale feature fusion strategies, and validates the contribution of each component through detailed ablation studies and interpretability analysis.

## 2. Related Work

### 2.1. Single-Branch Time Series Representation Learning Models

The core task of time series analysis lies in how to effectively extract and model temporal features in time series data. Traditional RNN [15] and CNN [16,17] methods process temporal dependencies and local features of time series through recurrent structures and convolution operations, respectively, but due to receptive field limitations, they face difficulties in capturing long-term dependencies. A Transformer can capture global dependencies through self-attention mechanisms, improving the modeling capability for temporal features in long sequences. However, a standard Transformer has high computational complexity and large memory consumption when processing long time series, becoming a bottleneck for applications. To address this issue, multiple optimization models have been successively proposed [18,19,20,21]. Additionally, Autoformer [22] introduces an auto-correlation mechanism that decomposes time series into long-term dependency and short-term variation components, enabling more effective modeling of periodic changes, reducing redundant computations, and improving efficiency. iTransformer [23] adjusts the self-attention mechanism through an inverted Transformer architecture, reducing computational complexity in long sequence processing while preserving the modeling capability for global temporal features. MICN [24] combines local and global features through a multi-scale modeling strategy, simultaneously capturing temporal features at different time scales to achieve more comprehensive feature learning. TCN [25] efficiently captures long- and short-term dependencies in time series data through convolution operations, particularly suitable for high-resolution short- to medium-term time series prediction, avoiding the gradient vanishing problem of traditional RNN models and demonstrating excellent performance in tasks with complex temporal features. PatchTST [26] optimizes computational and memory efficiency through subsequence segmentation and channel independence design while maintaining effective learning of local temporal features. These methods demonstrate the potential of learning representations in the temporal domain by improving long-term dependency modeling capability and computational efficiency in time series analysis.

### 2.2. Dual-Branch Time Series Representation Learning Models

Dual-branch models have clear advantages in time series analysis, especially when processing global and local features of time series data. For example, DCDetector [27] improves anomaly detection accuracy through dual attention mechanisms, Pathformer [28] addresses computational bottlenecks in long sequence modeling through improved self-attention mechanisms, and OneNet [29] effectively handles concept drift problems through online ensemble methods. Contiformer [30] proposes a flexible self-attention mechanism that adapts to irregular time intervals by weighting attention scores for each pair of time steps, effectively capturing long- and short-term dependencies while avoiding the impact of uneven sampling. ST-GNN [31] combines graph neural networks and spectral modeling, improving multivariate time series prediction accuracy through a dual-branch structure. Although these models have made progress on specific tasks, they still have limitations in computational efficiency and cross-task generalization capability. Beyond dual-branch designs, GAFormer [32] introduces instance-specific group embeddings to capture latent channel structures, while CCM [33] dynamically clusters channels based on intrinsic similarity to balance channel-independent and channel-dependent modeling. These works corroborate the value of adaptive grouping, which our learnable variable grouping strategy likewise exploits.

## 3. Results

### 3.1. Problem Definition

Given a multivariate time series $X\in {\mathbb{R}}^{T\times N}$, which contains T time steps and N variables, traditional Transformer-based methods mainly adopt time-centric attention mechanisms, treating each time step as the basic modeling unit and independently modeling the temporal dependencies of each variable. This approach faces computational complexity of $O(T^{2}\cdot N)$ when processing long sequences and cannot fully utilize the complex relationships between variables in multivariate systems. For prediction tasks, given historical observation values $X=x_{1},x_{2},\ldots ,x_{T}$, where $x_{t}\in {\mathbb{R}}^{N}$ represents the observations of all variables at the t−th time step, our goal is to predict the values for the next H time steps $Y=y_{1},y_{2},\ldots ,y_{H}$, where $y_{t}\in {\mathbb{R}}^{N}$.

### 3.2. Overall Framework

**Figure 2:**
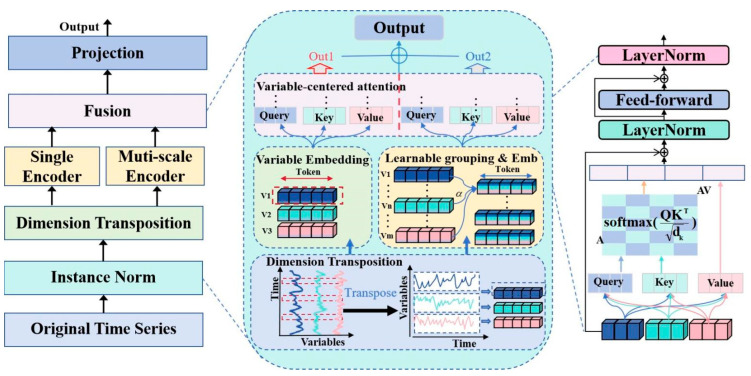
Overall framework of VCformer.

The core innovation of VCformer lies in shifting the attention paradigm from time-centric to variable-centric, while combining multi-scale variable grouping strategies to capture variable relationships at different granularities. The entire model adopts a dual-branch architecture that separately processes representation learning at the original variable level and grouped variable level, ultimately integrating multi-scale information through a fusion mechanism to generate prediction results. The model is illustrated in Figure 2.

The overall workflow of the model can be summarized into the following key steps: First, traditional time series inputs undergo transpose to make variables the primary modeling dimension; then, representations are learned for each variable through embedding layers; multi-scale representations are constructed using learnable variable grouping strategies; a dual-encoder architecture is employed to separately process original variable-level and grouped representations; finally, multi-scale information is integrated through an additive fusion mechanism to generate final predictions.

The entire model consists of two core modules: (1) Variable-Centric Multi-Scale Representation Learning Module, which is responsible for converting traditional time series into variable-centric representations and learning variable relationships at different granularities through multi-scale grouping strategies; (2) Dual-Branch Encoding and Fusion Module, which employs a dual-encoder architecture to separately process original variable-level and grouped variable-level representations, and integrates multi-scale information through fusion mechanisms.

### 3.3. Variable-Centric Multi-Scale Representation Learning Module

This module is the foundational component of VCformer, undertaking the key tasks of paradigm transformation and multi-scale representation construction. Traditional time-centric methods apply attention mechanisms to the temporal dimension, while our innovation lies in transferring this mechanism to the variable dimension, enabling the model to directly learn and utilize complex relationships between variables.

#### 3.3.1. Sequence Transposition and Variable Embedding

The first step of the model is to reorganize the dimensions of traditional time series input. We transpose the input time series $X\in {\mathbb{R}}^{T\times N}$ to $X^{T}\in {\mathbb{R}}^{N\times T}$, and this seemingly simple operation is actually the key to paradigm transformation, making each variable’s complete time series an independent processing unit and establishing variables as the primary modeling dimension. The transposed data undergoes feature extraction through variable embedding layers. The design of variable embedding layers considers that each variable may have different statistical characteristics and semantic meanings, therefore requiring specialized representation spaces to be learned for each variable:

(1) $$X_{embed}=\mathrm{VariableEmbed}(X^{T})=X^{T}W_{embed}+b_{embed}$$

where $W_{embed}\in {\mathbb{R}}^{T\times d_{model}}$ and $b_{embed}\in {\mathbb{R}}^{d_{model}}$ are embedding weight and bias parameters, respectively. This embedding operation maps original time series data to high-dimensional representation space $X_{embed}\in {\mathbb{R}}^{N\times dmodel}$, laying the foundation for subsequent inter-variable relationship modeling.

#### 3.3.2. Variable-Centric Attention Mechanism

The variable-centric attention mechanism is the core innovation of this method. Unlike traditional methods that independently compute cross-temporal attention for each variable, our method computes cross-variable attention for the entire time series, enabling the model to directly capture dependencies and mutual influences between variables. Given variable embeddings $X_{embed}\in {\mathbb{R}}^{N\times d_{model}}$, we obtain query, key, and value matrices through linear transformations. These matrices follow the standard Transformer architecture generation process, but their semantic meanings undergo fundamental changes:

(2) $$Q=X_{embed}W_{Q},\;K=X_{embed}W_{K},\;V=X_{embed}W_{V}$$

where $W_{Q},W_{K},W_{V}\in {\mathbb{R}}^{d_{\mathrm{model}}\times d_{k}}$ are learnable parameter matrices, and $d_{k}$ is the dimension of attention heads. In this setting, each row in the query matrix represents a query vector for a variable, and each row in the key matrix represents a key vector for a variable. Next, we apply multi-head attention mechanisms to compute the relationships between variables:

(3) $$\mathrm{VariableAttn}(Q,K,V)=A_{ij}V=\mathrm{softmax}\left(\frac{QK^{T}}{\sqrt{d_{k}}}\right)V$$

In this computation process, attention weights $A_{\mathrm{ij}}=\mathrm{softmax}\left(\frac{q_{i}k_{j}^{T}}{\sqrt{d_{k}}}\right)$ have clear physical meaning: they represent the importance degree of variable j for predicting variable i. This direct inter-variable attention computation enables the model to explicitly learn and utilize complex relationships between variables. For example, in traffic flow prediction, the flow of a certain road segment may be mainly influenced by adjacent segments and key nodes, and this dependency relationship can be directly reflected through attention weights.

#### 3.3.3. Learnable Variable Grouping Strategy

While variable-level attention can capture fine-grained relationships, real-world multivariate systems typically exhibit hierarchical structures. For example, in smart grids, variables can be grouped by geographical regions, voltage levels, or load types; in financial systems, stocks can be categorized by industry, market capitalization, or region. To capture such multi-scale relationship patterns, we introduce a learnable variable grouping strategy.

Given N variables and G groups, we define a learnable grouping matrix $W_{g}\in {\mathbb{R}}^{R\times N}$, where each row represents a variable group and each column corresponds to an original variable. To ensure the effectiveness and interpretability of grouping, we apply normalization to the grouping matrix:

(4) $$W_{g}^{\left(\mathrm{norm}\right)}=\mathrm{softmax}(W_{g})$$

The Softmax operation ensures that the contribution weights from each original variable to all groups sum to 1, maintaining information integrity while allowing variables to flexibly belong to multiple groups, reflecting the characteristic that variables in real systems may have multiple attributes.

The computation of grouped representations is based on weighted aggregation:

(5) $$X_{\mathrm{grouped}}=W_{g}^{\left(\mathrm{norm}\right)}X^{T}$$

where $X_{grouped}\in {\mathbb{R}}^{G\times T}$ represents grouped variable time series. Each group’s representation is a weighted combination of its member variables, with weights adaptively learned through end-to-end training. This design enables the model to automatically discover latent structures in data without requiring manual specification of grouping relationships between variables. In practical implementation, we also considered other grouping strategies for comparison, including adjacency-based grouping and overlapping sliding window grouping, but experimental results show that the learnable grouping strategy achieves optimal performance.

### 3.4. Dual-Scale Encoding and Fusion Module

After multi-scale representation learning, the model needs to perform deep feature extraction and effective integration of information at different scales. The dual-scale encoding and fusion module undertakes this critical task, transforming multi-scale information into unified predictive representations through specialized encoder architectures and fusion mechanisms.

#### 3.4.1. Dual-Scale Encoder Architecture

The key insight behind our dual-encoder design is that multivariate time series contain patterns at fundamentally different scales that require specialized processing. Fine-grained variable interactions (like the immediate influence between adjacent sensors) operate differently from coarse-grained group dynamics (like regional trends affecting multiple related variables). By using separate encoders, we allow each encoder to specialize in capturing these distinct types of dependencies. The original variable encoder acts as a “microscope,” focusing on detailed inter-variable relationships and local patterns that might be lost in aggregation. Meanwhile, the grouped variable encoder functions as a “telescope,” capturing broad systemic patterns and long-range dependencies that emerge at higher levels of abstraction. This division of labor allows each encoder to optimize for its specific scale of analysis.

**An original variable encoder** specifically processes fine-grained variable-level representations. It receives variable embeddings $X_{embed}$ as input and performs feature extraction through multiple variable-centric Transformer layers:

(6) $$H_{ori}={\mathrm{Encoder}}_{ori}(X_{embed})$$

Each layer follows the standard inverted Transformer structure, with attention mechanisms applied to the variable dimension:

(7) $$H^{\left(l+1\right)}=\mathrm{LayerNorm}(H^{\left(l\right)}+\mathrm{MultiHeadAttn}(H^{\left(l\right)}))$$

(8) $$H^{\left(l+1\right)}=\mathrm{LayerNorm}(H^{\left(l+1\right)}+\mathrm{FFN}(H^{\left(l+1\right)}))$$

where FFN is the feed-forward neural network and LayerNorm is the layer normalization operation. The original variable encoder can capture detailed relationships and local patterns between variables.

**A grouped variable encoder** processes coarse-grained group-level representations. It first embeds grouped variables and then performs feature learning through independent encoder layers:

(9) $$X_{group\_embed\;}=GroupEmbed(X_{grouped}\;)$$

(10) $$H_{group}={\mathrm{Encoder}}_{group}(\mathrm{Embed}(X_{grouped}))$$

The grouped encoder structure is similar to the original variable encoder but processes information at different scales. Through independent parameter learning, the grouped encoder can specifically capture macroscopic variable relationships and global patterns. The advantage of this dual-encoder design lies in, on one hand, the original variable encoder maintains sensitivity to fine-grained relationships. On the other hand, the grouped encoder reduces computational complexity through dimensionality reduction while focusing on learning high-level semantic relationships. The collaborative work of both encoders enables the model to understand and model multivariate time series at different abstraction levels. While the independent encoder design increases computational cost, it allows for specialized feature extraction for each scale.

#### 3.4.2. Multi-Scale Fusion Mechanism

The fusion mechanism addresses a fundamental challenge: how to combine insights from different analytical perspectives without losing the unique contributions of each scale. Our additive approach preserves the distinct information captured by each encoder while creating a unified representation that benefits from both fine-grained precision and macro-level understanding.

First, we need to address the dimensional mismatch between the two encoder outputs. Since the original variable encoder outputs $H_{ori}\in {\mathbb{R}}^{N\times dmodel}$ while the grouped variable encoder outputs $H_{group}\in {\mathbb{R}}^{G\times dmodel}$, we use linear interpolation to map grouped representations to the original variable dimensional space:

(11) $$H_{mapped}^{group}=\mathrm{Interpolate}(H_{group})$$

The fused representation is then computed as follows:

(12) $$H_{fused}=H_{ori}+H_{mapped}^{group}$$

The advantage of this additive fusion strategy lies in its simplicity and effectiveness. It can directly integrate information from two scales without introducing additional parameters while maintaining linear additivity of information. Experiments show that this simple fusion approach achieves good performance across multiple datasets.

To validate the effectiveness of the fusion strategy, we also implemented other fusion methods for comparison, including learnable weighted fusion, attention-based fusion, and gating fusion mechanisms. The specific forms of these methods are as follows:

Learnable weighted fusion adaptively adjusts weights for two scales by introducing learnable parameters:

(13) $$H_{fused}=\alpha H_{ori}+(1-\alpha )H_{group}^{interp}$$

The Gating Fusion Mechanism uses gating units to dynamically control information flow:

(14) $$g=\sigma (W_{g}[H_{ori};H_{group}^{interp}]+b_{g})$$

(15) $$H_{fused}=g⊙H_{ori}+(1-g)⊙H_{group}^{interp}$$

After multi-scale fusion, we map the fused representations to prediction ranges through linear projection layers:

(16) $$Y=H_{fused}W_{proj}+b_{proj}$$

where $Wproj\in {\mathbb{R}}^{dmodel\times H}$ and $b_{proj}\in {\mathbb{R}}^{H}$ are projection weights and biases, respectively. The final prediction output $Y\in {\mathbb{R}}^{N\times H}$ is obtained in standard format after transposition.

### 3.5. Complexity Analysis

VCformer has significant advantages in computational complexity compared to traditional methods. Traditional time-centric Transformer methods have an attention computation complexity of $O(T^{2}\cdot N)$, which becomes a performance bottleneck when processing long sequences. In contrast, our method transfers attention computation to the variable dimension, with the main computational complexity consisting of two parts: (1) original variable-level attention computation, $O(T^{2}\cdot N)$, and (2) grouped variable-level attention computation, $O(G^{2}\cdot d_{model})$.

Considering the computational cost of embedding layers $O(N\cdot T\cdot d_{model})$, the overall computational complexity can be expressed as $O(N\cdot T\cdot d_{model}+N^{2}\cdot d_{model}+G^{2}\cdot d_{model})$. In practical applications, since $d_{model}$ is typically a fixed model parameter, we can simplify this to $O(N\cdot T+N^{2}+G^{2})$. When sequence length T is much larger than the number of variables N (T >> N), this complexity offers significant advantages.

## 4. Experiments and Discussion

### 4.1. Datasets

To comprehensively evaluate the performance of the proposed method, we conducted experiments on seven widely used real-world multivariate time series datasets. These datasets cover different application domains and temporal frequencies with good representativeness. Detailed information is shown in Table 1.

ETT [34]: This dataset contains four subsets (ETTh1, ETTh2, ETTm1, ETTm2) that record temperature data from electrical transformers. ETTh1 and ETTh2 record data hourly, while ETTm1 and ETTm2 record data every 15 min. The data comes from two different electrical transformers, with each dataset containing seven feature dimensions.

Weather [35]: This dataset collected 21 meteorological indicators from the Max Planck Institute for Biogeochemistry weather station in Germany in 2020, including humidity, temperature, etc., with data recorded every 10 min.

Electricity [36]: This describes hourly electricity consumption data from 321 customers from 2012 to 2014, reflecting temporal patterns of electricity consumption and user differences.

Exchange [37]: The Exchange dataset records the exchange rate fluctuations between multiple countries’ currencies and the US dollar, exhibiting distinct time series characteristics and economic volatility patterns. The dataset contains daily frequency exchange rate data with an extended time span, effectively capturing exchange rate variations across different economic cycles.

### 4.2. Baseline Models

To fairly evaluate the performance of the proposed method, we selected seven representative time series forecasting models as baselines, covering methods from traditional approaches to the latest Transformer architectures.

Informer [34]: This is a classic Transformer-based time series forecasting model that addresses long sequence prediction problems through ProbSparse self-attention mechanisms and distillation operations, significant influencing the time series forecasting field.

Autoformer [22]: This model combines decomposition architecture with auto-correlation mechanisms, capable of progressively decomposing complex temporal patterns. By replacing traditional self-attention mechanisms with auto-correlation, it improves the ability to capture periodic patterns.

PatchTST [26]: This is a Transformer model based on a patch strategy that processes time series by segmenting them into patches, effectively reducing computational complexity and improving prediction performance.

iTransformer [23]: This model makes innovative improvements to traditional Transformer architecture by processing multivariate time series through dimension inversion, showing excellent performance in modeling inter-variable relationships.

TimeMixer [37]: This is a hybrid architecture model specifically designed for time series, combining multiple temporal modeling techniques with advantages in capturing complex temporal dependencies.

DLinear [38]: As a simple yet effective linear model, DLinear performs prediction through decomposition and linear mapping. Despite its simple structure, it performs excellently on many tasks, providing important performance benchmarks for complex models.

LightTS [39]: This is a lightweight time series forecasting model that significantly reduces computational overhead while maintaining prediction accuracy, suitable for resource-constrained application scenarios.

Experimental Setup: To ensure the fairness and comparability of the experimental results, all models adopt the same experimental settings, using MSE and MAE as evaluation metrics, setting multiple prediction lengths T ∈ {96, 192, 336, 720} for comprehensive evaluation, adopting standard train/validation/test set partitioning, with all experiments implemented under the PyTorch 2.0.0 framework to ensure result consistency. Through comparison with these representative baseline models, we can comprehensively validate the effectiveness and superiority of the proposed method across different datasets and prediction lengths.

### 4.3. Long-Term Forecasting

Table 2 shows a comprehensive comparison between VCformer and current state-of-the-art methods. The consistently superior performance of VCformer across all test scenarios is not accidental but stems from the correctness of its core design philosophy. Traditional time-centric methods focus attention on the temporal dimension, attempting to learn future trends from historical time steps, but this approach ignores an important fact: in multivariate systems, inter-variable relationships are often more stable and more predictive than individual variable temporal evolution patterns. Our variable-centric attention mechanism directly captures these cross-variable relationships, thereby achieving stronger predictive capability.

What is particularly noteworthy is that VCformer performs exceptionally well on high-dimensional datasets, showing outstanding performance on electricity datasets. This phenomenon reflects an important advantage of the variable-centric approach: as the number of variables increases, the interrelationships between variables become richer and more complex. Traditional time-centric methods struggle to effectively capture these relationships, while our approach can fully utilize this rich information by directly modeling the dependencies between variables.

Another striking finding is that as the prediction horizon increases, VCformer’s relative advantage becomes more pronounced. This phenomenon reveals the importance of variable relationships in long-term forecasting: while short-term temporal patterns may still be effective, as the prediction horizon extends, the reliability of these temporal patterns gradually decreases, whereas the structural relationships between variables provide more stable predictive signals.

This analysis highlights two key advantages of the VCformer architecture:

High-dimensional data handling: The variable-centric approach excels when dealing with many variables because it directly models inter-variable dependencies rather than trying to capture all relationships through temporal sequences.

Long-term prediction stability: Variable relationships tend to be more structurally stable over time compared to temporal patterns, making them more reliable for extended forecasting horizons.

### 4.4. Ablation Experiments

To validate the core value of VCformer’s dual-branch architecture, we designed targeted ablation experiments comparing three different model configurations: the complete dual-branch model VCformer, a single-branch model using only original variable sequences (without OV), and a single-branch model using only the Variable-Centric Multi-Scale Representation Learning Module (without MS). The experimental results are shown in Table 3. The experimental results clearly demonstrate the synergistic effect of the dual-branch architecture. Using the original sequence branch alone can capture fine-grained inter-variable relationships but lacks the ability to understand macroscopic patterns, while using only the multi-scale representation branch can discover high-level variable combination patterns but may lose important detailed information. The complete dual-branch model achieves a significant 12.4% performance improvement by effectively fusing two complementary representations, far exceeding the effect of single branches. The essence of this synergistic effect lies in the complementarity of information. Original variable-level information contains rich local features and subtle variations, which are crucial for capturing short-term fluctuations and anomaly patterns, while group-level information provides a system-level macroscopic perspective, helping to understand long-term trends and structural relationships. The combination of both enables the model to both “see the trees” and “see the forest,” thereby achieving more comprehensive and accurate predictions.

To validate our core hypothesis that variable-centric modeling yields superior forecasting performance compared to traditional time-centric approaches, we conducted a systematic ablation study. We applied sequence transposition to three established Transformer-based models: FEDformer, Reformer, and Informer. This allows us to isolate the impact of our paradigm shift while controlling for other architectural differences. The experimental results are shown in Table 4. The results demonstrate consistent improvements when adopting variable-centric input structures across all tested models and datasets. The key findings include the following: 1. All three baseline models (FEDformer, Reformer, Informer) show improved MSE and MAE scores when processing variable-centric inputs compared to their original time-centric implementations. 2. The improvement magnitude varies across datasets, with particularly notable gains on ETTh1 and ETTh2 datasets, suggesting that variable-centric modeling is especially beneficial for datasets with strong inter-variable dependencies. 3. The consistent improvements across different Transformer architectures (sparse attention in FEDformer, reversible layers in Reformer, sparse self-attention in Informer) demonstrate that the variable-centric paradigm provides fundamental modeling advantages that transcend specific architectural choices.

These results provide empirical validation for our core claim that treating variables as primary modeling entities, rather than time steps, leads to better capture of inter-variable relationships and improved forecasting accuracy.

### 4.5. Systematic Evaluation of Grouping Strategies

We designed three different grouping strategies to validate the effectiveness of our proposed learnable grouping method. The experimental results are shown in Table 5, clearly indicating that the learnable grouping strategy consistently outperforms fixed grouping methods. The underlying principle behind this phenomenon is that real-world variable relationships are often more complex and subtle than artificially designed simple rules. Learnable grouping allows the model to discover optimal variable clustering patterns based on the data’s own characteristics, thereby better capturing hidden inter-variable relationships. Although adjacent grouping is effective in certain cases (such as adjacent sensors in time series that may have spatial correlation), this heuristic approach cannot adapt to the characteristics of all datasets. For example, in stock market data, adjacent stock codes may belong to completely different industries, and their correlation may be much lower than stocks from the same industry scattered throughout the sequence. The overlapping grouping strategy shows performance between adjacent grouping and learnable grouping. While it increases model flexibility by allowing variables to participate in modeling multiple groups, it also introduces additional complexity and computational overhead. Experiments show that in most cases, learnable non-overlapping grouping can already sufficiently capture variable relationships, and the marginal benefit of the overlapping mechanism is limited.

### 4.6. Sensitivity Analysis and Optimal Configuration of Group Size

**Figure 3:**
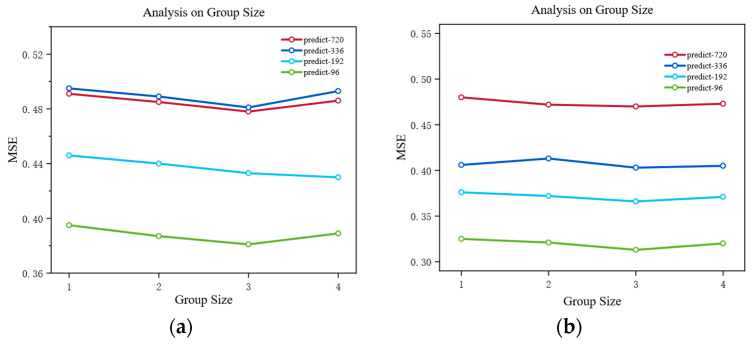
Sensitivity analysis of group size on model performance. The plots show the impact of different group sizes (1, 2, 3, 4) on forecasting performance across multiple prediction horizons. (**a**) The results of the ETTh1 dataset with prediction lengths of 96, 192, 336, and 720 steps. (**b**) The results of the ETTh2 dataset with the same prediction configurations. The MSE values are plotted against group sizes, with different colored lines representing different forecast horizons.

As shown in Figure 3, the choice of group size has a significant impact on VCformer’s performance, reflecting a core trade-off problem in multivariate time series modeling. Through systematic sensitivity analysis, we found that when the group size is set to 3, the model can maintain optimal or near-optimal performance across most prediction horizons. This phenomenon contains profound theoretical insights. The choice of group size is essentially about finding the optimal balance between information retention and pattern generalization. Specifically, groups that are too small can retain fine-grained information and local features of original variables to the greatest extent, but this detailed modeling approach may not effectively capture higher-level inter-variable collaborative relationships and macroscopic patterns. In this case, the model tends to fall into the trap of over-focusing on individual variable fluctuations while ignoring the collective behavioral characteristics of variable groups. Groups that are too large have the capability to discover macroscopic patterns and long-term trends, and can identify large-scale interactions between variables, but inevitably lose important fine-grained information during the information aggregation process. This information loss is particularly evident in short-term prediction tasks, as short-term forecasting often relies on precise capture of local variations. When the group size is 3, it is precisely at the optimal balance point of this trade-off. This configuration both retains sufficient detailed information to capture local changes and possesses sufficient abstraction capability to identify medium-scale relationship patterns between variables. From an information theory perspective, this group size can maintain information entropy while effectively reducing the interference of noise on model predictions.

### 4.7. Fine-Grained Analysis of Fusion Mechanisms

The effective fusion of multi-scale information is one of the key factors contributing to the success of VCformer. We designed three different fusion strategies, ranging from simple linear combinations to complex attention mechanisms, and systematically evaluated the effectiveness of various fusion methods. The experimental results are shown in Table 6.

Surprisingly, the simplest additive fusion strategy performed best or nearly best on most datasets. This phenomenon reveals a profound theoretical insight: when two information sources are both well-trained and contain highly complementary information, a simple linear combination is often sufficient. Additive fusion does not introduce any additional parameters, which not only significantly reduces the risk of overfitting but also makes the model easier to train and deploy. This advantage is particularly evident in scenarios with limited parameter resources or limited training data.

In contrast, while complex fusion mechanisms theoretically offer more refined information integration, the additional parameters may lead to overfitting, especially in small-sample learning scenarios. Concatenation fusion preserves the integrity of both information sources but may introduce information redundancy and dimensionality explosion issues; weighted fusion introduces learnable weights but may suffer from imbalanced weight distribution during the early stages of training, leading to the excessive suppression of one branch. Experiments show that when the two feature branches are sufficiently trained and well aligned, a simple addition operation can effectively integrate complementary information, while the marginal benefits of complex mechanisms are often offset by the training instability they introduce. This finding not only validates the rationality of the VCformer design but also provides important design principles for multi-scale information fusion tasks: under the premise of sufficient information complementarity, a simple design is often more effective than a complex architecture.

### 4.8. Visualization Experiments

**Figure 4:**
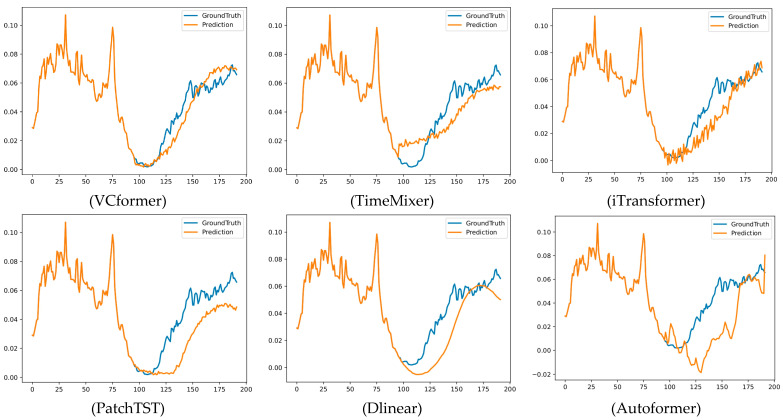
Visualization comparison of prediction performance across different models. The figure shows prediction curves comparing ground truth (blue) with model predictions (orange) for six different methods on the ETTh1 dataset with 96-step ahead forecasting. Each subplot displays the temporal evolution over approximately 200 time steps, demonstrating the models’ ability to capture trends, peaks, valleys, and local variations.

To more intuitively demonstrate VCformer’s prediction advantages, we designed prediction fitting curve comparison experiments. The visualization results are shown in Figure 4. This visualization analysis can not only validate the reliability of quantitative metrics but, more importantly, reveal the essential differences in prediction behavior between different models. By comparing the fitting curves of true values and predicted values, we can deeply understand each model’s prediction characteristics, error distribution patterns, and sensitivity to different types of changes. Compared to pure numerical indicators, visualization results can provide richer information dimensions, helping us identify performance differences in models in specific time periods or specific patterns, thereby providing more precise guidance for model improvement and application scenario selection.

From the comparison of prediction fitting curves, we can observe several important phenomena: **(1) Precise trend tracking**: VCformer demonstrates precise capability in capturing the overall trends of data, with prediction curves showing significantly higher overlap with true curves compared to baseline models, along with particularly outstanding performance at key nodes of long-term trend changes. **(2) Excellent peak/valley capture**: When data shows peaks and valleys and other critical turning points, VCformer can accurately predict the timing and magnitude of these extreme points, while baseline models such as TimeMixer and iTransformer often exhibit obvious lag phenomena or excessive smoothing problems, leading to the loss of critical information. **(3) Stable prediction consistency**: Throughout the entire prediction range, VCformer maintains consistent high-precision performance without obvious prediction degradation or increased fluctuation phenomena; this stability is crucial for practical applications. **(4) Detail fidelity advantages**: While maintaining overall trend accuracy, VCformer can also better preserve local fluctuation characteristics of the data, avoiding information loss caused by excessive smoothing, which reflects the unique advantages of its dual-branch architecture in multi-scale information.

### 4.9. Robustness Analysis

#### 4.9.1. Noise Robustness

**Figure 5:**
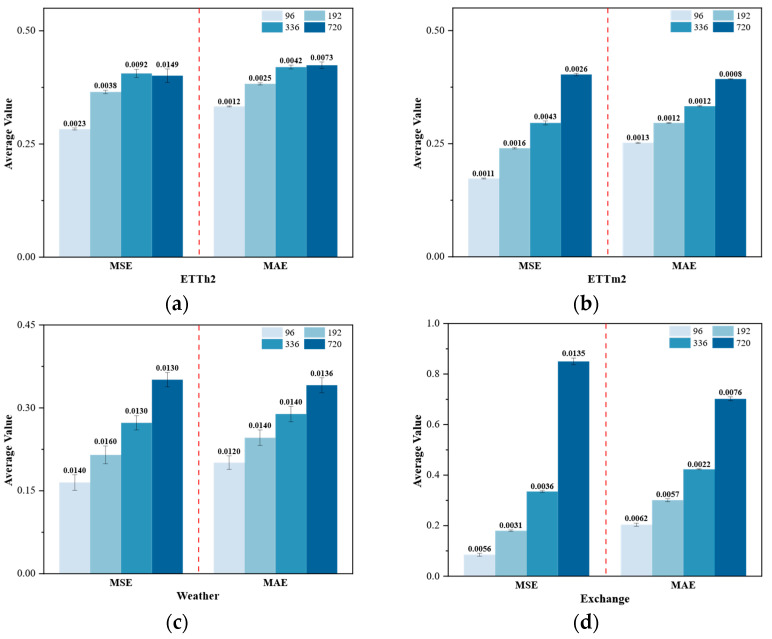
Robustness analysis under different noise levels. Performance comparison of VCformer across four datasets ((**a**) ETTh2, (**b**) ETTm2, (**c**) Weather, (**d**) Exchange) with noise injection at 5%, 10%, and 20% levels. Different bar colors represent different prediction horizons (96, 192, 336, and 720 steps). Results demonstrate model’s robustness with minimal performance degradation across varying noise conditions and forecast lengths. The red line divided the display of MAE and MSE.

Real-world data often contains noise and missing values, making model robustness crucial. We injected 5%, 10%, and 20% noise into the model, respectively, evaluating its performance differences in long-sequence prediction tasks. As shown in Figure 5, VCformer demonstrates excellent noise resistance capability, which stems from its variable-centric design philosophy. When individual variables are affected by noise interference, the model can compensate and correct through information from other related variables. This “collective wisdom” mechanism gives the model natural immunity to noise in individual variables.

From the experimental results, VCformer exhibits excellent stability under all noise levels. For the ETTh2 and ETTm2 datasets, even under 20% high-noise environments, the model performance degradation is relatively small, with MSE and MAE indicators showing significantly lower growth than expected. Particularly notable is that on more complex multivariate datasets such as Weather and Exchange, VCformer’s noise resistance advantages are even more prominent. This phenomenon can be explained from three perspectives: (1) Inter-variable information redundancy: Related variables often contain overlapping information, allowing other variables to provide supplementary information when some are contaminated by noise. (2) Robustness of the grouping mechanism: The learnable grouping strategy automatically identifies and aggregates strongly correlated variables, forming stable information units that maintain integrity even when individual variables exhibit anomalies. (3) Anti-interference capability of multi-scale feature fusion: The dual-branch architecture creates a multi-level information verification mechanism that effectively suppresses noise propagation between different scales. Experiments also show that VCformer’s noise resistance advantages become more apparent as prediction length increases, further confirming the importance and stability of variable relationships in long-term prediction.

#### 4.9.2. Parameter Sensitivity Analysis

To evaluate the robustness and generalizability of our model across different architectural configurations, we conduct a comprehensive sensitivity analysis on key hyperparameters. We focus on the impact of representation dimension D and the number of attention heads, as these are critical factors affecting model capacity and performance.

We evaluate VCformer’s sensitivity to representation dimensions D ∈ {128, 256, 512} across three datasets (ETTh1, ETTh2, Electricity). For each configuration, we conduct three independent runs with different random seeds to ensure statistical reliability. The number of attention heads is fixed at 8, following standard Transformer practices, while other hyperparameters remain consistent with our main experiments (batch size = 32, epochs = 10, learning rate explored in 10^−3^). Table 7 presents the sensitivity analysis results, showing the mean performance and standard deviation across three runs for each configuration.

The comprehensive robustness analysis presented in this section demonstrates that VCformer exhibits excellent stability across multiple dimensions: noise resistance, parameter sensitivity, and reproducibility. The model’s ability to maintain consistent performance under various perturbations stems from its variable-centric design philosophy, which creates inherent redundancy and cross-validation mechanisms through inter-variable relationships. This stability characteristic is particularly important for neural network architectures, as recent theoretical work has emphasized the critical role of global stability in ensuring the reliable performance of complex neural systems under various operational conditions [40]. Our empirical findings align with these theoretical insights, showing that architectural designs incorporating multi-scale information fusion and variable interdependencies naturally enhance system stability, making VCformer a robust solution for practical multivariate time series forecasting applications.

### 4.10. Performance Analysis

**Figure 6:**
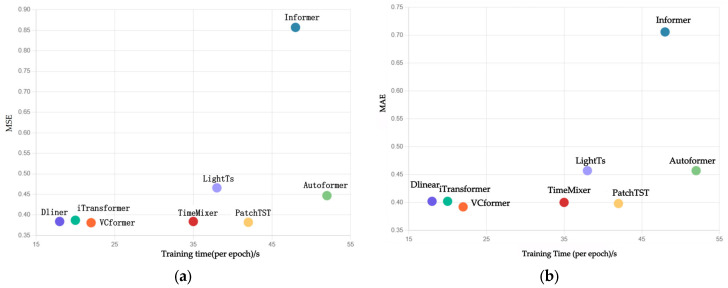
Comparison of training speed and MAE (**a**) and MSE (**b**) metrics with seven models.

This section evaluates the computational efficiency of VCformer compared to state-of-the-art baselines, with a focus on practical deployment considerations. Our dual-branch architecture introduces moderate computational overhead compared to single-branch models but maintains significant advantages over traditional time-centric Transformers. The analysis was conducted on NVIDIA A100 GPUs (NVIDIA, Santa Clara, CA, USA) using the ETTh1 dataset with input length L = 96. Figure 6a,b show the training speed and MAE and MSE metrics of VCformer compared to various baseline models on the ETTh1 dataset.

Our model VCformer adopts a variable-centric modeling paradigm, switching the modeling dimension from time to variables through sequence transposition, and introducing a dual-scale encoding structure to simultaneously capture interactions at the variable level and variable group level. Compared to traditional time-centric models, this structure has significant advantages in terms of prediction accuracy and interpretability. In contrast, DLinear is a lightweight linear model with an extremely simple structure, offering inherent advantages in terms of parameter scale and computational overhead, making it particularly suitable for resource-constrained or real-time-sensitive scenarios. While iTransformer also adopts a variable-centric modeling approach, it focuses solely on relationships between variables at a single scale, resulting in a lighter structure and lower computational complexity (O(N^2^)), outperforming our dual-scale structure in terms of efficiency. It is worth noting that traditional time-centric models based on the standard Transformer (such as Informer and Autoformer) have a computational complexity of O(T^2^) when the input sequence length is large, which is significantly higher than our O(N^2^). As the sequence length T increases, this gap will be further amplified, leading to a significant increase in training and inference costs.

## 5. Limitations and Future Directions

While VCformer demonstrates strong performance, several limitations warrant future investigation: (1) Scalability: The dual-encoder architecture increases computational overhead, requiring further optimization for extremely high-dimensional systems. (2) Dynamic relationships: Current grouping mechanisms assume relatively stable variable relationships, which may not capture rapidly evolving system dynamics. (3) Domain adaptation: The model’s transferability across different domains and the optimal configuration of hyperparameters require further study.

Future research directions include developing more efficient fusion mechanisms, exploring dynamic grouping strategies, and extending the framework to handle irregular time series and missing data scenarios. The variable-centric paradigm established in this work provides a solid foundation for advancing multivariate time series forecasting research and applications.

## 6. Conclusions

This paper proposes the Variable-Centric Transformer (VCformer), a novel multivariate time series forecasting framework that fundamentally shifts the modeling paradigm from time-centric to variable-centric through sequence transposition. The key innovation lies in recognizing that in multivariate systems, inter-variable relationships are often more informative and stable than individual temporal patterns for long-term prediction tasks. The variable-centric modeling approach successfully addresses the limitations of traditional time-centric methods by directly learning inter-variable dependencies, where each variable’s complete time series becomes an independent modeling unit with attention weights that possess clear physical interpretations. The proposed dual-scale processing architecture simultaneously captures fine-grained variable-level interactions and coarse-grained group-level collaborative patterns, achieving a significant 12.4% performance improvement compared to single-branch alternatives. Additionally, the learnable variable grouping strategy automatically discovers optimal variable clustering patterns without requiring prior domain knowledge, consistently outperforming fixed grouping methods while maintaining the computational complexity of O(N^2^) compared to O(T^2^) for traditional methods.

Comprehensive experiments on seven benchmark datasets demonstrate VCformer’s superior performance across multiple prediction horizons. The model achieves optimal or near-optimal performance on all datasets, with particularly strong advantages on high-dimensional data such as the Electricity dataset. The relative advantages become more pronounced as prediction horizons increase, validating the stability of variable relationships for extended forecasting. VCformer exhibits strong noise resistance capabilities with minimal performance degradation even under 20% noise conditions, while providing clear attention weight patterns that offer insights into inter-variable relationships and system dynamics. These experimental findings confirm the effectiveness of the variable-centric approach in capturing complex multivariate dependencies that traditional time-centric methods struggle to model effectively.

The variable-centric approach opens new possibilities for multivariate time series analysis, where the learned inter-variable attention weights serve as powerful tools for understanding system dynamics, identifying key variable relationships, and supporting decision-making processes. While VCformer demonstrates strong performance, future research should address scalability challenges for extremely high-dimensional systems, explore dynamic grouping strategies for rapidly evolving variable relationships, and investigate domain adaptation capabilities. The dual-scale architecture provides a flexible framework that can be adapted to various domain-specific requirements, and the variable-centric paradigm established in this work provides a solid foundation for advancing multivariate time series forecasting research and applications in fields ranging from financial markets to climate monitoring.

Future work will concentrate on “evolving variable relationships.” We will embed a lightweight, time-adaptive grouping module into VCformer that periodically revises variable clusters via sliding windows or gating updates, enabling the model to cope with abrupt or gradual changes in dependency structures common in energy and financial systems. Coupled with causal-discovery techniques, this extension will allow for the real-time identification and suppression of spurious correlations, ensuring long-term robustness and interpretability.

## Figures and Tables

**Table 1:** Overview of multivariate time series datasets used in experiments.

Dataset	Train	Valid	Test	Dim	Frequency
ETTh1, ETTh2	8545	2881	2881	7	Hour
ETTm1, ETTm2	34,465	11,521	11,521	7	15 min
Weather	36,792	5271	10,540	21	10 min
Exchange	5120	665	1422	8	Daily
Electricity	8545	2881	2881	7	Hourly

**Table 2:** Long-term forecasting performance comparison across different prediction horizons. The bold red represents the best effect, while the blue represents the second best.

Metric		VCformer		TimeMixer		iTransformer		PathchTST		LightTS		Dlinear		Autoformer		Informer	
		MSE	MAE	MSE	MAE	MSE	MAE	MSE	MAE	MSE	MAE	MSE	MAE	MSE	MAE	MSE	MAE
ETTh1	96	**0.381**	**0.392**	0.384	0.400	0.387	0.402	**0.382**	**0.398**	0.466	0.457	0.384	0.402	0.447	0.457	0.857	0.706
	192	**0.433**	**0.425**	**0.437**	**0.429**	0.438	0.437	0.441	0.433	0.510	0.476	0.435	0.434	0.503	0.485	1.004	0.781
	336	**0.481**	**0.439**	**0.473**	**0.437**	0.492	0.463	0.495	0.499	0.552	0.501	0.485	0.465	0.509	0.484	1.098	0.842
	720	**0.478**	**0.475**	0.586	0.446	0.502	0.490	**0.481**	**0.478**	0.587	0.543	0.517	0.523	0.492	0.501	1.185	0.856
	Average	**0.443**	**0.433**	0.470	**0.428**	0.455	0.448	**0.450**	0.452	0.529	0.494	0.455	0.456	0.488	0.482	1.036	0.796
ETTh2	96	**0.290**	**0.337**	0.297	0.348	0.299	0.347	**0.294**	**0.343**	0.417	0.446	0.471	0.382	0.344	0.382	0.954	0.775
	192	**0.370**	**0.388**	**0.369**	**0.392**	0.381	0.392	0.383	0.396	0.564	0.536	0.584	0.466	0.452	0.454	1.026	0.796
	336	**0.419**	**0.425**	0.427	0.432	0.421	0.433	**0.417**	**0.426**	0.671	0.577	0.816	0.531	0.473	0.476	1.033	0.782
	720	**0.421**	**0.439**	0.427	0.445	0.432	0.443	**0.421**	**0.437**	0.831	0.653	0.543	0.631	0.471	0.489	1.162	0.849
	Average	**0.375**	**0.397**	0.380	0.404	0.383	0.404	**0.379**	**0.401**	0.621	0.553	0.604	0.503	0.435	0.450	1.044	0.801
ETTm1	96	**0.313**	**0.343**	**0.320**	**0.357**	0.341	0.382	0.329	0.361	0.385	0.413	0.344	0.373	0.503	0.472	0.683	0.567
	192	**0.366**	**0.373**	**0.362**	0.382	0.385	0.394	0.371	0.385	0.410	0.425	0.382	0.391	0.543	0.497	0.794	0.659
	336	**0.403**	**0.399**	**0.396**	**0.406**	0.423	0.424	0.404	**0.406**	0.447	0.451	0.415	0.417	0.631	0.534	1.182	0.837
	720	0.470	**0.439**	0.468	0.445	0.477	0.467	**0.457**	**0.442**	0.523	0.509	0.469	0.454	0.651	0.571	1.151	0.824
	Average	**0.388**	**0.389**	**0.387**	**0.398**	0.407	0.417	0.390	0.399	0.441	0.450	0.403	0.409	0.582	0.519	0.953	0.722
ETTm2	96	**0.173**	**0.251**	0.180	0.259	0.183	0.275	**0.176**	**0.261**	0.221	0.325	0.191	0.295	0.258	0.341	0.369	0.456
	192	**0.239**	**0.295**	0.247	0.303	0.251	0.316	**0.243**	**0.305**	0.323	0.391	0.285	0.369	0.283	0.340	0.541	0.572
	336	**0.301**	**0.336**	0.307	0.339	0.318	0.353	**0.303**	0.343	0.436	0.459	0.371	0.423	0.337	0.372	1.342	0.857
	720	**0.401**	**0.394**	**0.396**	**0.399**	0.415	0.409	0.402	0.401	0.637	0.563	0.549	0.517	0.435	0.436	3.329	1.335
	Average	**0.279**	**0.319**	0.283	**0.325**	0.292	0.338	**0.281**	0.328	0.404	0.435	0.349	0.401	0.328	0.372	1.395	0.805
Weather	96	**0.164**	**0.196**	**0.168**	**0.209**	0.178	0.218	0.182	0.221	0.187	0.243	0.192	0.255	0.261	0.335	0.300	0.384
	192	**0.215**	**0.245**	**0.209**	**0.252**	0.224	0.259	0.226	0.261	0.231	0.294	0.235	0.296	0.307	0.367	0.578	0.534
	336	**0.272**	**0.287**	**0.274**	**0.293**	0.283	0.301	0.285	0.299	0.285	0.324	0.286	0.337	0.352	0.396	0.722	0.620
	720	**0.352**	**0.341**	**0.355**	**0.345**	0.357	0.354	0.359	0.348	0.354	0.361	0.346	0.383	0.419	0.428	0.834	0.736
	Average	**0.251**	**0.267**	**0.252**	**0.275**	0.261	0.283	0.263	0.282	0.264	0.306	0.265	0.318	0.335	0.382	0.609	0.569
Exchange	96	**0.086**	**0.204**	0.090	0.213	**0.087**	**0.206**	0.136	0.281	0.127	0.275	0.097	0.217	0.198	0.325	0.812	0.734
	192	**0.182**	**0.305**	0.188	0.315	**0.183**	**0.305**	0.238	0.374	0.236	0.377	0.185	0.316	0.300	0.366	1.116	0.825
	336	**0.329**	**0.417**	0.342	0.432	**0.338**	**0.420**	0.409	0.491	0.422	0.498	0.346	0.424	0.502	0.523	1.372	1.006
	720	**0.829**	**0.690**	0.868	0.721	0.853	0.697	0.866	0.711	0.925	0.752	**0.839**	**0.695**	1.449	0.934	2.448	1.195
	Average	**0.357**	**0.404**	0.372	0.420	**0.365**	**0.407**	0.412	0.464	0.428	0.476	0.367	0.413	0.612	0.537	1.437	0.940
Electricity	96	**0.150**	**0.236**	0.153	0.247	**0.149**	**0.243**	0.194	0.281	0.231	0.366	0.213	0.302	0.203	0.319	0.479	0.504
	192	**0.166**	**0.250**	0.170	**0.256**	**0.168**	0.257	0.196	0.234	0.237	0.339	0.216	0.306	0.225	0.337	0.630	0.606
	336	**0.178**	**0.264**	0.185	0.277	**0.176**	0.272	0.212	**0.255**	0.249	0.354	0.225	0.318	0.235	0.329	0.373	0.424
	720	**0.209**	**0.291**	0.225	0.310	**0.209**	**0.300**	0.254	0.334	0.290	0.377	0.268	0.351	0.255	0.363	0.410	0.420
	Average	**0.176**	**0.260**	0.183	0.273	**0.176**	**0.268**	0.214	0.276	0.252	0.359	0.231	0.319	0.230	0.337	0.473	0.489

**Table 3:** Ablation study results—comparison of complete VCformer with single-branch variants.

Model		VCformer		w/o MS Block		w/o OV Block	
Metric		MSE	MAE	MSE	MAE	MSE	MAE
ETTh1	96	0.381	0.392	0.387	0.402	0.395	0.411
	192	0.433	0.425	0.438	0.437	0.442	0.449
	336	0.481	0.439	0.492	0.463	0.488	0.457
	720	0.478	0.475	0.502	0.49	0.492	0.487
ETTh2	96	0.29	0.337	0.299	0.347	0.305	0.355
	192	0.37	0.388	0.381	0.392	0.387	0.403
	336	0.419	0.425	0.416	0.421	0.428	0.446
	720	0.421	0.439	0.432	0.443	0.441	0.452
Weather	96	0.164	0.196	0.178	0.218	0.172	0.209
	192	0.215	0.245	0.224	0.259	0.229	0.271
	336	0.272	0.287	0.283	0.301	0.289	0.315
	720	0.352	0.341	0.347	0.354	0.366	0.359
Exchange	96	0.086	0.204	0.087	0.206	0.084	0.199
	192	0.182	0.305	0.183	0.305	0.189	0.302
	336	0.329	0.417	0.338	0.42	0.325	0.422
	720	0.829	0.69	0.853	0.697	0.847	0.701

**Table 4:** Ablation study comparing variable-centric vs. time-centric input paradigms.

Metric		FEDformer		Reformer		Informer	
		MSE	MAE	MSE	MAE	MSE	MAE
ETThl	Original	0.383	0.424	0.826	0.715	0.857	0.706
	Invert	0.372	0.413	0.393	0.406	0.393	0.41
ETTh2	Original	0.341	0.388	2.626	1.155	3.654	1.536
	Invert	0.329	0.375	0.416	0.487	0.303	0.352
ETTml	Original	0.381	0.422	0.534	0.543	0.683	0.567
	Invert	0.369	0.403	0.331	0.366	0.334	0.372
ETTm2	Original	0.205	0.289	0.647	0.612	0.456	0.647
	Invert	0.193	0.279	0.179	0.263	0.18	0.263
Electricity	Original	0.189	0.312	0.369	0.424	0.479	0.504
	Invert	0.182	0.296	0.174	0.26	0.176	0.261
Exchange	Original	0.149	0.278	1.043	0.817	0.812	0.734
	Invert	0.133	0.259	0.189	0.21	0.088	0.21
Weather	Original	0.215	0.299	0.689	0.687	0.589	0.384
	Invert	0.209	0.282	0.179	0.22	0.163	0.206

**Table 5:** Performance evaluation of learnable vs. fixed grouping methods.

Strategy		Learnable Grouping		Adjacent Grouping		Overlapping Grouping	
Metric		MSE	MAE	MSE	MAE	MSE	MAE
ETTh1	96	0.381	0.392	0.398	0.412	0.388	0.401
	192	0.433	0.425	0.447	0.457	0.432	0.427
	336	0.481	0.439	0.499	0.486	0.486	0.445
	720	0.478	0.475	0.482	0.479	0.486	0.479
ETTh2	96	0.290	0.337	0.288	0.341	0.295	0.346
	192	0.370	0.388	0.379	0.396	0.367	0.381
	336	0.419	0.425	0.426	0.437	0.422	0.423
	720	0.421	0.439	0.427	0.436	0.425	0.447
Weather	96	0.164	0.196	0.181	0.248	0.169	0.199
	192	0.215	0.245	0.234	0.277	0.219	0.252
	336	0.272	0.287	0.285	0.312	0.277	0.285
	720	0.352	0.341	0.367	0.374	0.356	0.349
Exchange	96	0.086	0.204	0.090	0.203	0.088	0.201
	192	0.182	0.305	0.179	0.299	0.192	0.307
	336	0.329	0.417	0.348	0.433	0.333	0.415
	720	0.829	0.690	0.843	0.712	0.835	0.708

**Table 6:** Comparison of different fusion mechanisms for multi-scale information integration.

Fusion Method		Add		Concat		Weighted	
Metric		MSE	MAE	MSE	MAE	MSE	MAE
ETTh1	96	0.381	0.392	0.390	0.402	0.393	0.408
	192	0.433	0.425	0.439	0.442	0.442	0.439
	336	0.481	0.439	0.487	0.436	0.495	0.457
	720	0.478	0.475	0.485	0.483	0.499	0.488
ETTh2	96	0.290	0.337	0.298	0.346	0.298	0.351
	192	0.370	0.388	0.383	0.406	0.387	0.399
	336	0.419	0.425	0.427	0.433	0.423	0.429
	720	0.421	0.439	0.418	0.433	0.427	0.451

**Table 7:** Parameter sensitivity analysis of VCformer.

Datasets		ETTh1		ETTh2		Electricity	
Metric		MSE	MAE	MSE	MAE	MSE	MAE
D = 128	96	0.383 ± 0.001	0.392 ± 0.000	0.292 ± 0.000	0.341 ± 0.002	0.153 ± 0.001	0.239 ± 0.000
	192	0.431 ± 0.001	0.423 ± 0.001	0.370 ± 0.001	0.387 ± 0.001	0.169 ± 0.000	0.253 ± 0.002
	336	0.482 ± 0.003	0.443 ± 0.002	0.423 ± 0.002	0.428 ± 0.003	0.182 ± 0.002	0.269 ± 0.001
	720	0.479 ± 0.006	0.477 ± 0.007	0.422 ± 0.004	0.443 ± 0.000	0.208 ± 0.002	0.290 ± 0.000
D = 256	96	0.381 ± 0.000	0.392 ± 0.001	0.290 ± 0.003	0.337 ± 0.000	0.15 ± 0.001	0.236 ± 0.001
	192	0.433 ± 0.002	0.425 ± 0.003	0.370 ± 0.001	0.388 ± 0.003	0.166 ± 0.002	0.250 ± 0.001
	336	0.481 ± 0.003	0.439 ± 0.005	0.419 ± 0.000	0.425 ± 0.001	0.178 ± 0.001	0.264 ± 0.002
	720	0.478 ± 0.005	0.475 ± 0.006	0.421 ± 0.000	0.439 ± 0.002	0.209 ± 0.002	0.291 ± 0.001
D = 512	96	0.385 ± 0.000	0.394 ± 0.000	0.290 ± 0.001	0.339 ± 0.000	0.155 ± 0.001	0.238 ± 0.002
	192	0.437 ± 0.002	0.426 ± 0.004	0.371 ± 0.002	0.390 ± 0.001	0.170 ± 0.005	0.249 ± 0.001
	336	0.484 ± 0.005	0.440 ± 0.005	0.422 ± 0.001	0.427 ± 0.003	0.183 ± 0.002	0.269 ± 0.003
	720	0.477 ± 0.005	0.478 ± 0.008	0.420 ± 0.001	0.441 ± 0.000	0.215 ± 0.003	0.295 ± 0.005

## Data Availability

Data is contained within the article. The data presented in this study is available from the corresponding author upon reasonable request.
